# The Wiener–Hopf technique, its generalizations and applications: constructive and approximate methods

**DOI:** 10.1098/rspa.2021.0533

**Published:** 2021-10

**Authors:** Anastasia V. Kisil, I. David Abrahams, Gennady Mishuris, Sergei V. Rogosin

**Affiliations:** ^1^ Department of Mathematics, University of Manchester, Manchester M13 9PL, UK; ^2^ Isaac Newton Institute for Mathematical Sciences, University of Cambridge, Cambridge CB3 0EH, UK; ^3^ Aberystwyth University, Penglais, Aberystwyth SY23 3BZ, UK; ^4^ Belarusian State University, Nezavisimosti Avenue, 4, Minsk 220030, Belarus

**Keywords:** Wiener–Hopf, Riemann–Hilbert, factorization, partial indices, Riemann boundary value problem, applications

## Abstract

This paper reviews the modern state of the Wiener–Hopf factorization method and its generalizations. The main constructive results for matrix Wiener–Hopf problems are presented, approximate methods are outlined and the main areas of applications are mentioned. The aim of the paper is to offer an overview of the development of this method, and demonstrate the importance of bringing together pure and applied analysis to effectively employ the Wiener–Hopf technique.

## 1. Introduction

The Wiener–Hopf method has been motivated by interdisciplinary interests ever since its inception. It resulted from a collaboration between Norbert Wiener, who worked on stochastic processes, and Eberhard Hopf, who worked on partial differential equations (PDEs). The method was first described in their joint article [1] where they study a convolution-type integral equation for f(x) on a semi-axis

1.1 $$\int_{0}^{\infty }k(x-t)f(t)\text{d}t=g(x),\;x>0,$$

with k(x) and g(x) given.

The study of this equation was principally motivated by an interest of one of the authors, in a differential equation governing the radiation equilibrium of stars [2]. The Wiener–Hopf equation arises from extending the integral equation (1.1) into x<0,

1.2 $$\int_{0}^{\infty }k(x-t)f(t)\text{d}t=\left\{ \begin{array}{ll} g(x), & \text{for }x>0,\\ h(x), & \text{for }x\leq 0, \end{array}\right.$$

for some function h(x). Note that, although h(x) is unknown, it is not independent as it is uniquely defined by the left hand side of (1.2) once f(x) is determined. Applying the Fourier transform to (1.2) results in an equation of type (2.1) (the interaction of a Fourier transform with its convolution has to be used, and analytic properties of half-range Fourier transforms employed^1^). This laid the foundation for the study of scalar Wiener–Hopf equations.

Independently, the theory of the more general Riemann–Hilbert boundary value problem has been developed. The Riemann–Hilbert boundary value problem for a particular case was first formulated by Riemann as a part of the problem of construction of complex differential equation of the Fuchs type with a given monodromy. In the latter form, the problem was presented by Hilbert as his 21st mathematical problem for the twentieth century [4]. Plemelj [5] proposed a method of solution to the Riemann–Hilbert boundary value problem based on the reduction to a case of the homogeneous Riemann boundary value problem proving its solvability by means of the Fredholm alternative.^2^ An intensive study of the Riemann–Hilbert boundary value problem began in the 1930s. Properties of the factorization problem and its role in the study of the Wiener–Hopf integral equation were outlined in the seminal paper [6]. Further development of the factorization theory was described in the book by Litvinchuk & Spitkovskii [7]. Several known facts on factorization were presented in a recent survey [8] (see also [9]), where one can find, in particular, an extended list of references on the subject.

Simultaneously, the general theory of integral equations was developed intensively at the beginning of twentieth century. Of note are the contributions by Hilbert [10], Fredholm [11], Volterra [12] and Carleman [13]. The latter two authors also considered integral equations with kernels depending on the difference of arguments, but containing integrals with variable limits or along a finite interval, respectively. Nevertheless, the Wiener–Hopf integral equation (1.1) became an important independent subject of research due to its wide range of applicability and many connections. The Wiener–Hopf method has enabled researchers to solve analytically numerous previously intractable integral and partial differential equations, as well as many boundary value problems. To date, it still remains the standard method for solving a wide class of canonical physical problems. The method is an important cornerstone (among other methods) in areas of pure mathematics, for example in development of the abstract theory of singular linear operators, pseudo-differential operators, etc. Additionally, the exact solutions form the basis of approximate techniques applicable to more complicated problems. Moreover, even in case when the constructive (analytical) construction is available, it is not always possible to realize numerically with a confidence [14].

Recently, the Isaac Newton Institute for Mathematical Sciences, Cambridge, UK, organized a one-month research programme ‘Bringing pure and applied analysis together via the Wiener–Hopf technique, its generalizations and applications’, where the most important results in the area were presented and further developments of the method were considered [15]. This review was inspired by that programme. The aim of this paper is to show the beauty, principal features and perspectives of the Wiener–Hopf technique. The theory of scalar Wiener–Hopf equations is now very rich and well developed. In contrast, much less is known about matrix Wiener–Hopf equations. These are a natural extension of the scalar case, and enable us to model more advanced problems derived from applications [16]. As it stands, solutions to matrix Wiener–Hopf problems have to be constructed on a case-by-case basis, or in an approximate fashion; the main approaches will be outlined in this review. The Wiener–Hopf technique is currently used in a wide range of disciplines including acoustics, finance, Lévy processes, hydrodynamics, elasticity, potential theory and electromagnetism. As such, there are several disjoint communities who rely on the Wiener–Hopf method, and developments in one field sometimes go unnoticed in another. It is intended that this review helps to remove this artificial intradisciplinary and interdisciplinary boundary.

In summary, the Wiener–Hopf technique is captivating for many reasons:

— It enables us to solve numerous physical problems motivated by real world applications modelled, for example, by partial differential equations and stochastic processes.
— Subtle and diverse analytical methods are required to obtain solutions.
— Solutions of the Wiener–Hopf problems are remarkably revealing, thus often allowing for explicit evaluation of its asymptotic forms at all singular points and at infinity.
— Computing solutions to obtain practically useful results often requires involved and innovative numerical analysis.

This review offers a short introduction to the application, theory and numerical implementation of the Wiener–Hopf method; each element forms an important part of the whole.

The review has the following structure. The following section summarizes results related to the Wiener–Hopf technique, related equations and applications. Section 3 lists the main constructive methods for certain classes of matrix Wiener–Hopf equations allowing for explicit factorization. Section 4 outlines several approximate methods available for the cases where exact constructive methods do not apply. The last section draws conclusions and discusses further directions.

## 2. Preliminaries

### (a) The Wiener–Hopf problem

We begin by formulating the basic Wiener–Hopf equation (resulting from applying the Fourier transform to equation of type (1.2)) and discussing the standard method of its solution (for details of its derivation, see [17]). This will be compared and contrasted with the Riemann–Hilbert boundary value problem at the end of the next section. The Wiener–Hopf equation has a simpler form and historically was the first to be discovered, so forms a natural starting point.

Define *upper half-plane* $H^{+}=\{\text{Im}\;z>a;a<0\}$, *lower half-plane* $H^{-}=\{\text{Im}\;z<b;b>0\}$ and *the Wiener–Hopf strip* $H=H^{+}\cap H^{-}=\{a<\text{Im}\;z<b\}$ (a and b are real numbers, typically small). Then the classic *Wiener–Hopf problem* is, for given K(z) and C(z) analytic in H, and

2.1 $$K(z)\Phi_{+}(z)+\Psi_{-}(z)+C(z)=0,\;z\in H,$$

to find $\Phi_{+}(z)$ an unknown function analytic in $H^{+}$ and $\Psi_{-}(z)$ an unknown function analytic in $H^{-}$. The function K(z) is called *the Wiener–Hopf kernel corresponding to (2.1)*.

For the sake of completeness, we are going to outline the usual procedure used to solve the Wiener–Hopf problem in the case of canonical scalar factorization. The key step in the Wiener–Hopf procedure is to find $K_{+}$ and $K_{-}$ (*the Wiener–Hopf factors*) such that

2.2 $$K(z)=K_{+}(z)K_{-}(z),\;z\in H.$$

with $K_{\pm }$ and their inverses analytic in $H^{\pm }$. This product is referred to as a *factorization of the kernel* and is the most important part of the procedure; it is straightforward to accomplish for scalar kernels.

Multiplying through by $K_{-}^{-1}$ we obtain

2.3 $$K_{+}\Phi_{+}(z)+K_{-}^{-1}\Psi_{-}(z)+K_{-}^{-1}C(z)=0,\;z\in H.$$

Now performing an *additive splitting* we have

2.4 $$K_{-}^{-1}C(z)=C_{-}(z)+C_{+}(z),\;z\in H,$$

where $C_{\pm }(z)$ are analytic in their indicated half-planes $H^{\pm }$, respectively. Finally, rearranging the equation yields

2.5 $$K_{+}\Phi_{+}(z)+C_{+}(z)=-K_{-}^{-1}\Psi_{-}(z)-C_{-}(z),\;z\in H.$$

The right-hand side of (2.5) is a function analytic in $H^{+}$ and the left-hand side of equation (2.5) is analytic in $H^{-}$. Hence, together they offer analytic continuation from H into the whole of the z-plane, and so each side is equal to an entire function J(z), say. We also require *at most algebraic growth* of each side of equation (2.5) as |z|→∞ in the respective half-planes of analyticity, i.e.

2.6 $$J(z)\leq O(|z{|}^{n}),\;|z|\to \infty ,$$

for some constant n. Applying the extended form of Liouville’s theorem implies that J(z) is a polynomial of degree less than or equal to n. Hence $\Phi_{+}(z)$ and $\Psi_{-}(z)$ are determined up to ⌊n⌋+1 unknown constants (typically n≤0 or 1, and the constants can be fixed using extra information about the behaviour of the solution). Then the application of the inverse Fourier transform to $\Phi_{+}(z)$ or $\Psi_{-}(z)$ would yield the solution to (1.1).

If $\Phi_{+}(z)$, $\Psi_{-}(z)$ and C(z) in (2.1) are vectors of functions with a matrix of functions K(z), then (2.1) is called *matrix/vectorial Wiener–Hopf equations*. All the above steps in the solution are analogous. The difficulty lies in performing the multiplicative factorization (2.2), which becomes the main challenge to overcome and is addressed in §§3 and 4.

### (b) Riemann–Hilbert boundary value problem

The Wiener–Hopf equation is closely related to the Riemann–Hilbert boundary value problem^3^ [18,45]. The latter problem is to determine two functions or vectors of functions $\Phi^{\pm }(z)$, which are analytic in the complementary domains $D^{\pm }$ (i.e. $D^{+}\cup L\cup D^{-}=C$, with a given simple closed curve $L=\partial D^{+}=\partial D^{-}$) and satisfy on L the following linear condition:

2.7 $$\Phi^{+}(t)=G(t)\Phi^{-}(t)+g(t),\;t\in L,$$

with the function/matrix G(t) and the function/vector g(t) given on L.

In the scalar case, the complete solution of the Riemann problem (2.7) was given by Gakhov [18]. He has shown that solvability of the problem depends on the *Cauchy index* (winding number) of the coefficient G

2.8 $$\kappa ={\text{ind}}_{L}G=\frac{1}{2\pi }\int_{L}\text{d}(\arg G(t)).$$

In the case of Hölder-continuous functions G and g, a simple smooth contour L and in the case of non-negative index κ≥0 the solution of (2.7) has the form^4^

2.9 $$\Phi^{\pm }(z)=X^{\pm }(z)[\Psi^{\pm }(z)+P_{\kappa }(z)],\;z\in D^{\pm },$$

where $P_{\kappa }(z)$ is a polynomial of order κ, and

2.10 $$\begin{array}{rl} & X^{+}(z)=\exp\{\Gamma^{+}(z)\},\;X^{-}(z)=\exp\{z^{-\kappa }\Gamma^{-}(z)\}, \end{array}$$

2.11 $$\begin{array}{rl} & \Gamma^{\pm }(z)=\frac{1}{2\pi i}\int_{L}\frac{\log[t^{-\kappa }G(t)]\text{d}t}{t-z},\;z\in D^{\pm }, \end{array}$$

2.12 $$\begin{array}{rl} & \Psi^{\pm }(z)=\frac{1}{2\pi i}\int_{L}\frac{g(t)}{X^{+}(t)}\frac{\text{d}t}{t-z},\;z\in D^{\pm }. \end{array}$$

In the case of negative index κ, the unique solution has the form $\Phi^{\pm }(z)=X^{\pm }(z)\Psi^{\pm }(z)$ provided that a finite number of solvability conditions

$$\int_{L}\frac{g(t)}{X^{+}(t)}t^{k-1}\text{d}t=0,\;k=1,2,\ldots ,-\kappa -1,$$

are satisfied (otherwise there is no solution).

The scalar and matrix function factorization, related to the Riemann–Hilbert boundary value problem, was introduced by Birkhoff [19] and also in a more straightforward form by Gakhov [20]. Factorization means obtaining the following representation of an n×n matrix function G, given on a bounded curve L (curve such that $0\in D^{+}$, $\infty \in D^{-}$), as

2.13 $$G(t)=G^{+}(t)\Lambda (t)G^{-}(t),\;t\in L,$$

where $G^{\pm }(t)$ are boundary values of bounded analytic zero-free matrices $G^{\pm }(z),z\in D^{\pm }$, and

2.14 $$\Lambda (t)=\text{diag}\{t^{\kappa_{1}},t^{\kappa_{2}},\ldots ,t^{\kappa_{n}}\},$$

where $\kappa_{j}\in Z,j=1,2,\ldots ,n$, are integer numbers called *partial indices*. Representation (2.13) is called a *left-sided factorization*. Interchanging $G^{+}$ and $G^{-}$ we arrive at the *right-sided factorization*. Respectively, the partial indices are called left (right) partial indices. In general, they are not the same; however, their sum is always equal to the index of det G (this can sometimes fail for functions with severe discontinuities worse than the piece-wise continuous case [9], theorem 3.21). In the case L=R the variable t in (2.14) is replaced by the ratio (t−i)/(t+i) (similar changes are need in (2.10) and (2.11)). In this case one needs to assume the continuity of G at infinity.

Unfortunately, in the general case, there exists no method of determining the partial indices, while such information is important to reconstruct the factors, $G^{\pm }$, and is instrumental in proving the stability of any respective algorithm. Those issues are discussed further in §4. Factorization, i.e. determination of the partial indices $\kappa_{j}\in Z,j=1,2,\ldots ,n$, and factors $G^{+},G^{-}$ is an independent problem playing a crucial role in many theoretical and applied problems.

We would like to make here a few comments on formal differences between the Wiener–Hopf and Riemann–Hilbert problem. Note that (2.13) is different to (2.2) since equation (2.2) does not contain Λ(t). It is because it is common in the Wiener–Hopf literature [17] to implicitly assume that κ=0 in formulae (2.10) and (2.11), which is often the case in applications. On the other hand, (2.13) can be always presented in the form (2.2) by further factorizing the matrix $\Lambda (t)=\Lambda^{+}(t)\Lambda^{-}(t)$, and then redistributing the respective diagonal terms. However, this destroys some of the assumptions on the behaviour of the factors $G^{\pm }$: the behaviour at infinity, satisfaction of the invertibility property (i.e. it introduces zeros into the half-planes of analyticity), etc. Thus, this form of the factorization is tightly linked to the conditions imposed on the factors (which space of analytic function is chosen) [7]. Note also that the factors $K_{\pm }$ themselves, (2.2), would in general have non-zero partial indices. Another important difference between the approaches is that (2.2) is valid on a strip (and thus, knowledge of the possible poles and zeros inside the strip provides useful information for solving the problem under consideration), while the factorization (2.13) is defined on a line (that can be analytically extended if possible). These differences are also discussed in [21]. Finally, we would like to stress that there are authors associating the Wiener–Hopf problem with the Riemann–Hilbert one and vice versa that only underlines the fact that the division is rather artificial and depends on the research communities. To avoid any unintentional misleading, we discuss particular factorization techniques below as they have appeared in the original presentations, with minor comments where necessary.

### (c) Canonical matrix

The factorization problem (2.13) is almost equivalent to the homogeneous (i.e. with g(t)≡0) Riemann–Hilbert boundary value problem (2.7):

2.15 $$\Phi^{+}(t)=G(t)\Phi^{-}(t),\;t\in L.$$

In [22], the conditions for the existence of a solution to (2.15) are described. If the solution to (2.15) exists one can find^5^ a so-called *fundamental system of solutions* $\Phi_{1}(z),\ldots ,\Phi_{n}(z)$ and the corresponding *fundamental matrix* $\Phi (z)=(\Phi_{1}(z),\ldots ,\Phi_{n}(z))$ with vectors $\Phi_{j}(z)$ being their columns. Fundamentality of the system/matrix means that the determinant of the fundamental matrix cannot be identically zero.

The next step (which is more constructive [22, p. 520]) is to transform the fundamental system of solutions to a normal form. By definition the *normal system of solutions* $\Psi_{1}(z),\ldots ,\Psi_{n}(z)$ to (2.15) is the fundamental system such that the determinant of the fundamental matrix does not vanish anywhere on C (including the curve L). The matrix $\Psi (z)=(\Psi_{1}(z)\ldots \Psi_{n}(z))$ of the normal system is called the *normal matrix*.

By elementary transformations, the normal system of solutions can be transformed to the so called *canonical system*. The canonical system is usually denoted $X_{1}(z),\ldots ,X_{n}(z)$ and its matrix is $X(z)=(X_{1}(z)\ldots X_{n}(z))$. The properties of the canonical system are

(i) The canonical system is the normal system of solutions to (2.15), i.e. the determinant of the canonical matrix det X(z) is nowhere vanishing in C.
(ii) The sum of the orders $\kappa_{j}$ at infinity of the columns of the matrix X(z) (i.e. solutions $X_{j}(z)$) is equal to the Cauchy index of the determinant det X(z).

They are called partial indices of the Riemann–Hilbert boundary value problem (2.15). The integer numbers $\kappa_{j}$ are the same as partial indices of the factorization (2.13) of the matrix function G(t) (the coefficient in (2.15)).

Whenever the canonical matrix is constructed, the solution of factorization problem (2.13) is presented in the form

2.16 $$G^{+}=X^{+},\;G^{-}=\Lambda^{-1}{\left(X^{-}\right)}^{-1},$$

where $\Lambda (z)=\text{diag}\{z^{\kappa_{1}},\ldots ,z^{\kappa_{n}}\}$ (this applies when $0\in D^{+}$).

Note that for a canonical matrix it is said that the matrix $X^{-}(z)$ has *the normal form at infinity* for a factorization (2.13). An example of the use of this method is presented in §3(c).

### (d) Other convolution-type integral equations

Related to the integral equations of the convolution type (1.1) on the half-axis are paired equations

2.17 $$\begin{array}{rl} & f(x)+\int_{-\infty }^{\infty }k_{1}(x-t)f(t)\text{d}t=g(x),\;x>0\\ \text{and}\; & f(x)+\int_{-\infty }^{\infty }k_{2}(x-t)f(t)\text{d}t=g(x),\;x<0, \end{array}\}$$

and the so-called transpose to the dual equations:

2.18 $$f(x)+\int_{0}^{\infty }k_{1}(x-t)f(t)\text{d}t+\int_{-\infty }^{0}k_{2}(x-t)f(t)\text{d}t=g(x),\;x\in R.$$

They can be both solved in a very similar manner to (1.1) by extending the range, taking the Fourier transform of each of the equations, and eliminating one unknown, to give rise to the usual Wiener–Hopf equation

2.19 $$F^{+}(s)=R(s)F^{-}(s)+T(s),\;R(s)=\frac{1+K_{2}(s)}{1+K_{1}(s)},$$

where $K_{j}(s)$ are Fourier transforms of the kernels $k_{j}(x)$, j=1,2, and $F^{\pm }$, T(s) are different for each equation (details can be found in [18,23]).

### (e) Carleman-type boundary value problem

A closely related equation, but much less known is the so-called smooth-transition equation

2.20 $$f(x)+\int_{-\infty }^{\infty }k_{1}(x-t)f(t)\text{d}t-g(x)+{\text{e}}^{-x}\left\{f(x)+\int_{-\infty }^{\infty }k_{2}(x-t)f(t)\text{d}t-g(x)\right\}=0,$$

for −∞≤x≤∞. The interesting feature of this equation is that for x large and positive it is close to

2.21 $$f(x)+\int_{-\infty }^{\infty }k_{1}(x-t)f(t)\text{d}t-g(x)=0,\;x\gg 1,$$

And for x large and negative it is close to

2.22 $$f(x)+\int_{-\infty }^{\infty }k_{2}(x-t)f(t)\text{d}t-g(x)=0,\;x\ll -1,$$

which is similar to (2.17). Finally, for values of x=0 close to zero, the equation takes the form:

2.23 $$2f(x)+\int_{-\infty }^{\infty }k_{1}(x-t)f(t)\text{d}t+\int_{-\infty }^{\infty }k_{2}(x-t)f(t)\text{d}t-g(x)=0,\;|x|\ll 1.$$

This analysis explains the name of the equation; it behaves as the dual equation when |x|≫1 and as the transpose to the dual when |x|≪1.

In order to analyse this equation, define the following [3]

2.24 $$\phi (x)=f(x)+\int_{-\infty }^{\infty }k_{2}(x-t)f(t)\text{d}t-g(x),\;-\infty \leq x\leq \infty .$$

Then the original equation (2.20) can then be written as

2.25 $$f(x)+\int_{-\infty }^{\infty }k_{1}(x-t)f(t)\text{d}t-g(x)+{\text{e}}^{-x}\phi (x)=0.$$

After application of Fourier transform to (2.24) and (2.25) (again, with a capital letter indicating the transform of a lower-case letter) we arrive at the following equations

2.26 $$\begin{array}{rl} \Phi (z) & =(1+K_{2}(z))F(z)-G(z),\;-\infty \leq z\leq \infty \\ \text{and}\;-\Phi (z+i) & =(1+K_{1}(z))F(z)-G(z),\;-\infty \leq z\leq \infty . \end{array}\}$$

Eliminating F(z) we arrive at

2.27 $$(1+K_{1}(z))\Phi (z)=-(1+K_{2}(z))\Phi (z+i)+(K_{2}(z)-K_{1}(z))G(z),\;-\infty \leq z\leq \infty .$$

This is not a standard form for a Riemann–Hilbert boundary value problem but it can be transformed to one by defining a new function

2.28 $$w(\psi )=\frac{1}{\sqrt{\psi }}\Phi \left(\frac{\ln(\psi )}{2\pi }\right),$$

then the jump in the Riemann–Hilbert boundary value problem is across the cut argψ=0.

The smooth transition equation (2.20) is a special case of a more general Carleman-type boundary value problem: to find Φ(z) analytical in a domain D and satisfy on part of the boundary of D denoted S the following condition:

2.29 Φ(t)=E(t)Φ(m(t))+g(t),t∈S,

with the functions E(t), m(t) and g(t) given on S ([3], §15.1). Also it is required that m(t) is continuous and maps to the rest of the boundary of D. Additionally we require that t and m(t) transverse the boundary in different directions. Sometimes its solution can be reduced to a Riemann–Hilbert boundary value problem, but in general no closed-form solution is known [24]. Many applied problems are reduced to certain types of functional-difference equation (related to Wiener–Hopf and Riemann–Hilbert problems). We mention here the problems of applied mechanics [25–29] and diffraction theory [30–32].

### (f) Discrete Wiener–Hopf equation

In this section, we will look at an infinite system of equations of Wiener–Hopf type, a discrete analogue of integral equation (1.1). For all infinite sequence $a_{n}$ considered here we will assume the following holds for some constant M

2.30 $$|a_{n}|<\frac{M}{n^{1+\lambda }},\;0<\lambda <1.$$

We will also assume that the Fourier series $A(\theta )=\sum_{n=-\infty }^{\infty }a_{n}{\text{e}}^{\text{i}n\theta }$ is Hölder continuous.

The *discrete Wiener–Hopf* equations for $x_{n}$ is

2.31 $$\sum_{k=0}^{\infty }a_{n-k}x_{k}=c_{n},\;n=0,1,2\ldots ,$$

where the infinite sequences $a_{n}$ and $c_{n}$ are given. This is called an infinite system with a Toeplitz operator due to a presence of n−k in the index of a. Similar to the integral equation case, we first need to extend this equation for n negative

2.32 $$\sum_{k=0}^{\infty }a_{n-k}x_{k}=\left\{ \begin{array}{ll} c_{n} & \text{for }n=0,1,2,\ldots \\ d_{n} & \text{for }n=-1,-2,\ldots \end{array}\right.$$

for some $d_{n}$, which are unknown for n=−1,−2…. Note that the $d_{n}$ are uniquely defined by the left-hand side of (2.32) once $x_{k}$ is determined. Next we apply the discrete analogue of Fourier transform, the Z-transform. The Z-transform of a sequence $a_{n}$ is defined as

2.33 $$A(t)=\sum_{k=-\infty }^{\infty }t^{k}a_{k},$$

and as before is denoted by a capital letter. We multiply the nth equation in (2.32) by $t^{n}$ and sum over all equations:

2.34 $$\sum_{n=-\infty }^{\infty }t^{n}\sum_{k=0}^{\infty }a_{n-k}x_{k}=\sum_{n=0}^{\infty }t^{n}c_{n}+\sum_{n=-1}^{-\infty }t^{n}d_{n}.$$

The main property is the interaction of the Z-transform with a Töplitz operator

2.35 $$\sum_{n=-\infty }^{\infty }t^{n}\sum_{k=0}^{\infty }a_{n-k}x_{k}=\sum_{k=0}^{\infty }t^{k}x_{k}\sum_{n=-\infty }^{\infty }t^{n-k}a_{n-k}=\sum_{k=0}^{\infty }t^{k}x_{k}\sum_{m=-\infty }^{\infty }t^{m}a_{m},$$

which corresponds to the Fourier transform changing a convolution into a product. Thus, via a Z-transform, equation (2.32) becomes

2.36 $$A(t)X^{+}(t)=C(t)+D^{-}(t),$$

which holds on the unit circle, and the superscript +/− indicates the function is analytic inside/outside the unit circle. This Riemann–Hilbert boundary value equation can be solved as before to find $X^{+}(t)$ and then the inverse of the Z-transform applied to recover $x_{n}$.

A slightly more general infinite system which can be solved in an identical fashion is the discrete version of equation (2.17)

2.37 $$\begin{array}{rl} \sum_{k=-\infty }^{\infty }a_{n-k}x_{k}=c_{n}, & \;\text{for }n=0,1,2,\ldots \\ \text{and}\;\;\sum_{k=-\infty }^{\infty }b_{n-k}x_{k}=d_{n}, & \;\text{for }n=-1,-2,\ldots \end{array}\}$$

where $a_{k}$, $b_{k}$, $c_{k}$ and $d_{k}$ are known. Another version of the discrete equation is the analogue to the transpose to the dual equation (2.18):

2.38 $$\sum_{k=0}^{\infty }a_{n-k}x_{k}+\sum_{k=-1}^{-\infty }b_{n-k}x_{k}=c_{n},\;n=0,\pm 1,\pm 2,\ldots$$

Another interesting discrete system which has an analytic solution via a Wiener–Hopf technique is

2.39 $$\sum_{k=-\infty }^{\infty }a_{n-k}x_{k}-c_{n}+r^{n}\left(\sum_{k=-\infty }^{\infty }a_{n-k}x_{k}-c_{n}\right)=0,\;n=0,\pm 1,\pm 2,\ldots ,$$

where |r|≠ 1. With the help of a Z-transform, this can be reduced to a boundary value problem of Carleman type [3].

There are other methods for solving equation of type (2.31) without the use of Wiener–Hopf method. The discrete systems of equations with a Toeplitz operators have effective numerical methods of solutions, some which use Cauchy-like matrices, which have small numerical ranks [33]. Also there are some results about perturbation of Toeplitz operators for example solution of infinite systems like Au+Bu=f where A is Toeplitz operator and B is in some sense small [34].

Equations of type (2.31) naturally appear in applications when the boundary conditions are discrete and periodic in semi-infinite configurations. For example, this happens in the Sommerfeld half plane problem for discrete Helmholtz equation [35]. This is also the case when an semi-infinite discrete array of point scatterers is considered [36–39]. Additionally, this type of problems is common in crack propagation problems [40].

### (g) Non-uniqueness of factorization

This section gives the details of the degree of freedom when constructing Wiener–Hopf factorizations. In the scalar case, factors are unique up to a constant. In other words, if there are two such factorizations $K(z)=K_{+}(z)\lambda (z)K_{-}(z)$ and $K(z)=P_{+}(z)\lambda (z)P_{-}(z)$, where λ(z) is the factor accounting for the index of the function K(z),

2.40 $$K_{+}=cP_{+}\;\text{and}\;K_{-}=c^{-1}P_{-},$$

where c is some non-zero complex constant. This can be seen by applying analytic continuation to $K_{+}/P_{+}$ and $P_{-}/K_{-}$ and then using the extended Liouville’s theorem [41]. Here we deal with factorization of the matrix defined on a simple closed curve L in the complex plane. The corresponding result in the 2×2 matrix case is rather more complicated:

**Theorem 2.1. [9]**

*Let the matrix* G(t) *admit the Wiener–Hopf factorization* (2.13). *Then*:

2.41 $$\text{G}={\text{G}}_{+}^{1}\Lambda {\text{G}}_{-}^{1},$$

*where*

2.42 $${\text{G}}_{+}^{1}={\text{G}}_{+}\text{H},\;{\text{G}}_{-}^{1}=\Lambda^{-1}{\text{H}}^{-1}\Lambda {\text{G}}_{-},$$

*is also a factorization of* G(t) *where* H *is a constant invertible matrix function if* $\kappa_{1}=\kappa_{2}$ *and otherwise is of the form*:

2.43 $$\text{H}(t)=\left(\begin{array}{cc} c_{1} & P(z)\\ 0 & c_{2} \end{array}\right),\;z=\frac{t-i}{t+i},$$

*where* P(z) *is a polynomial of degree* $\kappa_{1}-\kappa_{2}$ *in* z.

This means that there is more freedom to choose the Wiener–Hopf factors when $\kappa_{1}\neq \kappa_{2}$. In the case of higher order matrix functions, similar results can be shown, while the matrix structure has a bit more complicated form [42].

### (h) General class of PDEs that can be reduced to a Wiener–Hopf

As an illustration of the concept, we consider a class of partial differential equations (PDEs) that can be reduced to a Wiener–Hopf equations or a Riemann–Hilbert boundary value problem ([3], §20.3). This method is suitable for linear PDEs of the form

2.44 $$\sum_{p=0}^{A}\sum_{q=0}^{B}h_{pq}(y)\frac{\partial^{p+q}u(y,x)}{\partial y^{p}\partial x^{q}}=g(y,x),$$

where $h_{pq}$ and g are given and u is to be determined; note that $h_{pq}$ has no x dependence. In the simplest case, the boundary conditions are given on the lines y=const. or half lines y=const. for x>0 or x<0. Let these n lines be positioned at constants $y_{1},\ldots ,y_{n}$. The boundary conditions have the form

2.45 $$\begin{array}{rl} & \sum_{p=0}^{P}\sum_{q=0}^{Q}\left(a_{pqrs}(y)\frac{\partial^{p+q}u(y_{s}+0,x)}{\partial y^{p}\partial x^{q}}+b_{pqrs}(y)\frac{\partial^{p+q}u(y_{s}-0,x)}{\partial y^{p}\partial x^{q}}\right)\\ & \;=g_{rs}(x),\;x>0\\ & \sum_{p=0}^{P}\sum_{q=0}^{Q}\left(c_{pqrs}(y)\frac{\partial^{p+q}u(y_{s}+0,x)}{\partial y^{p}\partial x^{q}}+d_{pqrs}(y)\frac{\partial^{p+q}u(y_{s}-0,x)}{\partial y^{p}\partial x^{q}}\right)\\ & \;=g_{rs}(x),\;x<0 \end{array}$$

2.46 $$\begin{array}{rl} \text{and}\; & r=1,\ldots m_{s},\;\;s=1,\ldots n. \end{array}$$

There could also be some conditions for y→∞ or y→−∞. In order to obtain a Riemann–Hilbert boundary value problem a list of systematic steps is performed in detail in ([3], §20.3), and which is translated into English in [43].

We also refer an interested reader to the classic monographs, for example, [17,45,44] for applications of various integral transforms to reduce PDE boundary value problems to Wiener–Hopf or Riemann–Hilbert form, among others.

### (i) Applications

The wide applicability of the Wiener–Hopf methods has ensured its continuous development from its inception to modern day. Traditional applications areas are acoustics, aeroacoustics, water waves, electromagnetism [46–49] as well as Lévy processes and signal processing [50–53]. Various static and dynamic problems of fracture mechanics [54–58], lattices [59–65], metamaterials and biomechanics are studied using this technique [40,66–69].

The Wiener–Hopf method is extensively used for canonical scattering problems both in acoustics [17] and electromagnetism [49]. In both of these applications, the governing equation is Helmholtz’s equation. There are fewer methods available to tackle Helmholtz’s equation compared to Laplace’s equation. For the Laplace equation, other techniques from complex analysis such as conformal mapping are applicable [70]. Although the Helmholtz equation is self-adjoint addition of boundary conditions, including the Sommerfeld radiation condition, means that the boundary value problem is no longer self-adjoint. This causes problems in demonstrating completeness of series expansions [71]. The Wiener–Hopf method on the other hand very naturally incorporates the radiation condition and complicated edge conditions. Simple models in aeroacoustics can also be reduced to acoustic problems via Lighthill’s analogy and the reciprocal theorem [72].

Water waves is an area where the Wiener–Hopf techniques is accepted as one of key analytic tools [73]. The ice cover on water can be modelled as an elastic material for which flexural motions are able to be described by thin-plate or Timoshenko–Mindlin theory [74–76]. Eigenfunction-matching method is also a commonly used method and in some cases this has been shown to be equivalent to the Wiener–Hopf method [77,78]. Recently, the problem of waves generated in a fluid and an ice sheet by a pressure region moving on the free surface of the fluid, along the edge of the semi-infinite ice sheet, has been solved using the Wiener–Hopf technique [79].

One of the new applications of the Wiener–Hopf technique has been nanophotonics. Graphene and other two-dimensional (2D) materials may sustain evanescent, fine-scale electromagnetic waves that are tightly confined to the boundary, called plasmonpolariton [66]. This is also closely related to edge magnetoplasmons, the theory of which was also derived using the Wiener–Hopf method [80] and the more realistic extension of which lead to more complicated Wiener–Hopf systems [81]. Recently, composites and metamaterials have also been investigated using the Wiener–Hopf method [36,67,82,83].

This is by no means an exhaustive list of applications; unfortunately space constrains us in this short survey. We now list a number of approaches to particular constructive exact or approximate factorization methods in the sections below.

## 3. A list of constructive procedures

By constructive factorization, we mean that there is an algorithmic way to obtain the factorization (2.13) or (2.2). Recall, for scalar functions, factorization is always possible via integrals of Cauchy type (2.11) and (2.12). Currently, there are no constructive factorization procedures for matrix functions in general, and only very specific classes can be exactly solved. What complicates the development of an algorithmic approach is that for a given matrix function it is difficult to determine if any known constructive procedure apply. Pre- and post-multiplication can sometimes transform the matrix to a known form, but this is undertaken mainly using *ad hoc* techniques. A systematic treatment of some transformations is given in [84].

The main classes of matrix kernels which have a constructive factorization are reviewed below. They are the rational matrix functions, commutative matrices, particular matrices with exponential factors, as well as several miscellaneous classes. We also refer the reader to several other important classes of piece-wise constant matrix functions not included in the review [42,85,86], the Meister and Speck matrix as discussed in [87] and for applications [88,89]. We also do not consider the case where there are singularities on the curve L [90]; for more information, the reader is refereed to [91,92].

### (a) Rational matrix function

This is the simplest class of matrix functions for which an exact constructive factorization is known. This is because there are only a finite number of isolated singularities which need to be considered. A procedure to construct the Wiener–Hopf factorization is known in this case and is briefly described immediately below, followed by the standard ‘pole removal’ technique.

#### (i) Factorization of rational matrix functions

This subsection describes an algorithm to factorize a rational matrix function ([9], §1.2). Given a matrix function M it can be expressed as P/q, where P is a polynomial matrix function and q is a scalar polynomial. It is enough to factor them separately. The scalar function q can be factorized easily, see §2(b). We will assume that det P has no roots on the real axis. What prevents a polynomial matrix P being a plus factor is the presence of zeros of det P in the upper half-plane. (Recall that, for factorization, a plus (minus) factor requires the matrix *and* its inverse to be analytic in the upper (lower) half plane.) A way of eliminating one zero at a time is outlined in the following lemma.

**Lemma 3.1.**

*Let* P *be a polynomial matrix function such that* det P *has no roots on the real axis. If* det P *has* n *roots in the upper half-plane, then we can construct a polynomial* L=PR *where* det L *has* n−1 *roots in the upper half-plane and the matrix* R *has the form (blanks stand for zeroes)*:

3.1 (1  −c1z−z0   1 ⋮    ⋱−ck−1z−z0     1z−z0      ⋱      1),

*where* $z=z_{0}$ *is one of the aforementioned poles, while* $c_{k}$ *are computed constructively and uniquely*.

We can repeat this procedure, eliminating all the roots of det P in the upper half-plane and hence construct the plus factor. Note that the matrix R is invertible. The minus factor can be found either by pre-multiplying P by the inverse of the plus factor, or by taking the inverse of the product of all of the R matrices. This completes the factorization.

#### (ii) ‘Pole removal’ for rational matrix functions

In applications the above rational factorization algorithm is rarely used; instead ‘pole removal’ or ‘singularity matching’ is employed [49,93] and is recapped below.

Suppose that A(z) is rational (every entry of the matrix function is a rational function), then we can solve (2.1) without multiplicative factorization. Perform an additive split $A(z)\Phi_{+}(z)={\left(A(z)\Phi_{+}(z)\right)}^{-}+{\left(A(z)\Phi_{+}(z)\right)}^{+}$ and $C(z)=C^{-}(z)+C^{+}(z)$, where the first term is analytic in $H^{-}$ and second in $H^{+}$. Since A(z) is rational we can write ${\left(A(z)\Phi_{+}(z)\right)}^{-}=\sum_{i=0}^{n}(A_{i}/\left(z-z_{i}\right))$, where $z_{i}$ are the poles in the upper half-plane, and $A_{i}$ are constants (residues at $z_{i}$). Thus,

3.2 $$A(z)\Phi_{+}(z)-\sum_{i=0}^{n}\frac{A_{i}}{z-z_{i}}+C^{+}(z)=-\Psi_{-}(z)-\sum_{i=0}^{n}\frac{A_{i}}{z-z_{i}}-C^{-}(z)$$

separates terms on the left- and right-hand sides into functions analytic in the upper and lower half planes respectively. To find $A_{i}$ we need to solve a linear system of equations (by setting $z=z_{i}$).

The pole removal method has an advantage over the rational factorization procedure. It enables one to remove undesired poles without changing the rest of the equations. This is one of the reason that it can not only be applied to rational Wiener–Hopf kernels but also to more general kernels which have one or more rational parts. Pole removal is closely linked to the generalized Liouville’s theorem (which allows the unknown function to have poles). It can also be naturally extended to meromorphic functions by allowing an infinite sum, which would lead to an infinite linear system that would need to be truncated in order to be solved. The additional advantage of ‘pole removal’ is that the multiplicative factorization is reduced to an additive splitting. It is also worth noting that many alternative formulations, such as Fredholm factorization [49,94,95], approximate techniques (see §4) and numerical methods [96], are also based on this principle or replacing a multiplicative factorization by additive splittings.

**Remark 3.2.**

Note that the instability issue discussed in §4 still applies here. To recognize the challenge it is enough to refer to the fact that, generally speaking, all poles or zeros are computed approximately, while the system of linear equations mentioned above (which have to be solved to compute the constants) can be ill-conditioned. In the case of a stable set of the partial indices the system is not ill-conditioned, otherwise, at least for some of the poles, this would be expected [97].

### (b) Analytic and meromorphic matrix functions

In [98] the factorization problem for the meromorphic matrix function was solved. The corresponding algorithm is based on the reduction of the problem to a finite number of systems of linear algebraic equations, whose coefficients’ matrices are block Toeplitz matrices:

3.3 $$T_{k}=\left(\begin{array}{cccc} C_{k} & C_{k-1} & \cdots & C_{-2\kappa }\\ C_{k+1} & C_{k} & \cdots & C_{-2\kappa +1}\\ \cdots & \cdots & \cdots & \cdots \\ C_{0} & C_{-1} & \cdots & C_{-2\kappa -k} \end{array}\right),$$

with $C_{j}$ being the power moments of the inverse $G^{-1}(t)$ to the given matrix with respect to the contour L

3.4 $$C_{j}=\frac{1}{2\pi i}\int_{L}t^{-j-1}G^{-1}(t)\text{d}t.$$

The partial indices of the left and right factorization are represented in terms of ranks of the above Toeplitz matrices. An analogous method is applied in [99] for the case of analytic matrix functions which, being a special case of the latter, need less computation. A part of this algorithm related to factorization of the scalar polynomial is implemented in the Maple computer system as module PolynomialFactorization (see its description and illustrative examples in [100]).

### (c) Triangular matrix functions

Upper or lower triangular matrix functions (with factorizable diagonal elements) can sometimes be reduced to a set of scalar equations. If the equations can be solved in turn and the previous solution used to make the next equation scalar, then the system decouples. However, even in the case of triangular matrix functions there may be complications which mean that the system does not decouple in this way. This happens when the partial indices are non-zero, for which case we describe a general procedure below. It relies on the idea of a canonical matrix, introduced in §2(c) and this was one of the first attempts to realize this construction explicitly (i.e. to determine canonical matrix factorization for (2.15)). The classical results Chebotarev [101] applies to 2×2 triangular matrix functions with factorizable diagonal entries.

Let

$$G(t)=\left(\begin{array}{cc} \zeta_{1}(t) & 0\\ a(t) & \zeta_{2}(t) \end{array}\right),\;t\in T\;\text{(unit circle)}.$$

Denote $\kappa_{j}={\text{ind}}_{\Gamma }\zeta_{j}(t)$ and let $x_{j}^{\pm }(z)$ be canonical functions for the scalar homogeneous Riemann–Hilbert boundary value problems with coefficients $\zeta_{j}(t)$, respectively [18], j=1,2. Then the piece-wise analytic matrix

$$X^{\pm }(t)=\left(\begin{array}{cc} x_{1}^{\pm }(t) & 0\\ x_{2}^{\pm }(t)\phi^{\pm }(t) & x_{2}^{\pm }(t) \end{array}\right)$$

with

$$\phi^{\pm }(z)=\frac{1}{2\pi i}\int_{T}\frac{a(\tau )x_{1}^{-}(\tau )\text{d}\tau }{x_{2}^{+}(\tau )(\tau -z)},\;z\in D^{\pm },$$

satisfies the following boundary condition: $X^{+}(t)=G(t)X^{-}(t).$ Let μ≥1 be the order of the function $\phi^{-}(t)$ at infinity. The orders of non-zero elements of the matrix $X^{-}(z)$ at infinity can be characterized by the following table:

$$\left(\begin{array}{cc} \kappa_{1} & -\\ \kappa_{2}+\mu & \kappa_{2} \end{array}\right).$$

If $\kappa_{1}\leq \kappa_{2}+\mu$, then the matrix $X^{-}(z)$ has the normal form at infinity, i.e. it is the canonical matrix. Thus, partial indices of the matrix G(t) are equal $\left(\kappa_{1},\kappa_{2}\right)$. In the case $\kappa_{1}>\kappa_{2}+\mu$, Chebotarev proposed to use an expansion of $1/\phi^{-}(z)$ as a continued fraction

$$\frac{1}{\phi^{-}(z)}=q^{\gamma_{0}}(z)+\frac{1}{q^{\gamma_{1}}(z)+(1/\left(q^{\gamma_{2}}(z)+\ldots \right))},$$

where $q^{\gamma_{i}}(z)$ are polynomials of orders $\gamma_{i}$, respectively ($\gamma_{0}=\mu$). Using these polynomials in the scheme of the elementary transformations it was shown in [101] that in a finite number of steps the ‘minus’-matrix can be rebuilt to the normal form at infinity and thus the partial indices will be found and factors in (2.13) will be determined. In [102], this approach was applied to obtain an explicit solution of the factorization problem for a case of the Khrapkov–Daniele matrix functions. In [103], this approach was applied to solve R-linear conjugation problem.

In [104] Chebotarev’s approach was realized for triangular matrix function of arbitrary order with factorizable diagonal entries. The basis for the inductive steps is the following statement: *let* L be a simple smooth closed contour 0∈L, and let B(t), for t∈L, be a non-singular Hölder continuous square matrix-function of order n having the following form:

3.5 $$B(t)=\left(\begin{array}{cc} A(t) & \text{0}\\ b_{1}(t)\ldots b_{n-1}(t) & c(t) \end{array}\right),\;\text{0}=\left(\begin{array}{c} 0\\ \vdots \\ 0 \end{array}\right).$$

*Suppose that the non-singular square matrix-function* A(t) *of the order* n−1 *admits the factorization*

$$A(t)=A^{+}(t)\;\Lambda (t)\;A^{-}(t),$$

*where* $\Lambda (t)=\text{diag}\;\{t^{\kappa_{1}},\ldots ,t^{\kappa_{n-1}}\}$. *Then the matrix-function* B(t) *possesses a factorization if the following matrix does:*

3.6 $$\left(\begin{array}{cc} \Lambda (t) & \text{0}\\ \left(\text{b}(t)|{\text{Y}}_{1}^{-}(t))\ldots (\text{b}(t)|{\text{Y}}_{n-1}^{-}(t)\right) & c(t) \end{array}\right).$$

*Here* $\text{b}(t)=(b_{1}(t),\ldots ,b_{n-1}(t))$ *is the row of the first* n−1 *entries of the lowest row of* B(t), ${\text{Y}}_{j}^{-}(t)={\left(y_{1j}^{-}(t),\ldots ,y_{(n-1)j}^{-}(t)\right)}^{T}$ *is the* jth *column of the matrix-function* $Y^{-}(t)={\left(X^{-}(t)\right)}^{-1}$*, and*

$$(\text{b}(t)|{\text{Y}}_{j}^{-}(t))=\sum_{k=1}^{n-1}b_{k}(t)y_{kj}^{-}(t).$$

This process can be repeated n times.

Note that for a matrix with a specific structure, the general stability criterion for the partial indices can be extended (in other words, within a specific sub-algebra, there are cases when the perturbation preserving the structure remains stable, while there exists a general (small) perturbation of other structure that changes the set of the partial indices. For the 2×2 triangular matrices such analysis has been conducted in [97].

### (d) Functionally commutative matrix functions

There has been significant progress in the case of the matrices possessing commutative factorizations [7]. One of the reasons is that one can apply some of the techniques that have been developed in the scalar setting. We present the corresponding result in the case of Hölder continuous coefficients G and g in (2.7).

In [105], the following question was considered: whether there exist a class of matrix coefficients G(t) such that formula (2.9) still gives the bounded solution of the vector-matrix problem (2.7) (i.e. for n>1)? Such a question is related to the purely algebraic question of the fulfilment of the identity

3.7 $${\text{e}}^{A(t)}{\text{e}}^{B(t)}={\text{e}}^{A(t)+B(t)},$$

for certain matrices A(t) and B(t) connected to the coefficient G(t). It was shown in [105] that identity (3.7) holds (and thus Gakhov’s formula (2.9) can be used to solve problem (2.7) in the matrix case), whenever the matrix function G(t) is functionally commutative. The latter condition means

3.8 G(t)G(τ)=G(τ)G(t),∀ t,τ∈L.

The question of solvability of the vector-matrix problem (2.7) in the case of a functionally commutative matrix G(t) is solved in [105]. In [106], the following necessary and sufficient condition for a matrix to be functionally commutative is reported: *the matrix* G(t) *is functionally commutative if and only if it can be represented in the form*

3.9 $$G(t)=\varphi_{1}(t)G_{1}+\varphi_{2}(t)G_{2}+\cdots +\varphi_{m}(t)G_{m},$$

*where* $\varphi_{1}(t),\varphi_{2}(t),\ldots ,\varphi_{m}(t)$ *are linear scalar independent functions, and* $G_{1},G_{2},\ldots ,G_{m}$ *are constant linear independent mutually commutative matrices.* The structure of lower-dimensional (up to n=4) functionally commutative matrix functions was described in [106] too. In [107] a solution to the Riemann–Hilbert boundary value problem (2.7) and the corresponding factorization problem is illustrated by examples [108].

### (e) Commutative factorization

Close to the above case is so called commutative factorization. Let us formulate it in the Wiener–Hopf setting. Let the matrix K(z) be defined on a strip H={z∈C:a<Im z<b} around the real line. The *commutative factorization* of K(z) are factors $K_{-}(z)$ and $K_{+}(z)$ satisfying (2.2) and the factors $K_{+}$, $K_{-}$ commute in H. The idea of commutative factorization stems from the work of Heins [109]. For criteria when commutative factorization is possible, the reader is referred to [110]. Several special cases of matrices admitting commutative factorizations are described below.

#### (i) Khrapkov–Daniele matrices

Matrices of this kind represent one of the simplest classes of matrices with commutative factorization. In 2×2 case, these matrices were discovered from certain diffraction problems [111–113]. They appear in the study of diffraction by geometries which had a right angle such as wedges or gratings (a grating can be thought of as a perpendicular strip in a waveguide) [114] as well as other configurations [46,115–118]. The Khrapkov–Daniele matrices have the following form

3.10 $$K(\alpha )=k_{0}(\alpha )I+k_{1}(\alpha )J(\alpha ),$$

where J(α) is a polynomial matrix-function with the property

3.11 $$J^{2}(\alpha )=\Delta^{2}(\alpha )I,$$

with $k_{0},k_{1}$ being arbitrary functions analytic in H and having algebraic growth at infinity and $\Delta^{2}$ is a polynomial in α,

$$\Delta^{2}(\alpha )=O(|\alpha {|}^{p}),\;\alpha \to \infty ,$$

with p≤2.

Commutative factorization under the above conditions has the form [116]

3.12 $$K^{\pm }(\alpha )=r^{\pm }(\alpha )\exp\{\theta^{\pm }(\alpha )J\},$$

where scalar functions $r^{\pm },\theta^{\pm }$ satisfy the relations

3.13 $$r^{+}(\alpha )r^{-}(\alpha )=r(\alpha ):=\sqrt{k_{0}^{2}(\alpha )-\Delta^{2}(\alpha )k_{1}^{2}(\alpha )}$$

and

3.14 $$\theta^{+}(\alpha )+\theta^{-}(\alpha )=\theta (\alpha ):=\frac{1}{\Delta (\alpha )}\tanh^{-1}\left(\frac{k_{1}(\alpha )}{k_{0}(\alpha )}\Delta (\alpha )\right).$$

An interesting approximate technique, based on the Khrapkov–Daniele matrix form, which allows it to be extended to certain non-commutative matrices, has also been developed [119].

#### (ii) Jones class

Jones [120] established the commutative factorization of the following n×n class of matrices

3.15 $$C(\alpha )=\sum_{m=1}^{n}a_{m}(\alpha )E^{m}(\alpha ),$$

where $a_{1}(\alpha ),a_{2}(\alpha ),\ldots ,a_{n}(\alpha )$ are functions analytic in the strip H, E(α) is an entire matrix-function, $E^{n}=q^{n}(\alpha )I$. It is supposed also that det C(α)≠0 and $\text{tr}\;E^{r}=0,r=1,2,\ldots ,n-1$.

Under these conditions, it was shown that C(α) possesses the commutative factorization

$$C(\alpha )=C^{+}(\alpha )C^{-}(\alpha )=C^{-}(\alpha )C^{+}(\alpha ),$$

where

3.16 $$\begin{array}{rl} & C^{\pm }(\alpha )={\left[{\left(\det\;C(\alpha )\right)}^{\pm }\right]}^{1/n}\sum_{p=0}^{n-1}\gamma_{p}^{\pm }(\alpha )E^{p}(\alpha ), \end{array}$$

3.17 $$\begin{array}{rl} & \gamma_{p}^{\pm }=\sum_{m=0}^{n-1}\frac{\omega^{mp}}{nq^{p}}\exp\left(\sum_{r=1}^{n-1}q^{n-r}\omega^{rm}\delta_{n-r}^{\pm }\right), \end{array}$$

3.18 $$\begin{array}{rl} \text{and}\;\; & \delta_{s}^{+}+\delta_{s}^{-}=\delta_{s}:=\sum_{p=0}^{n-1}\frac{\omega^{sp}}{nq^{s}}\ln\left[\sum_{l=1}^{n}q^{n-l}\omega^{lp}a_{n-l}\right]. \end{array}$$

Here $\omega ={\text{e}}^{(2\pi \text{i})/n}$ and the functions $\delta_{s}^{+},\delta_{s}^{-}$ are analytic in the corresponding half-planes.

In [116], the Wiener–Hopf factorization is constructed for matrices of the Jones class under assumption that E is an entire matrix-function with polynomial elements having distinct eigenvalues $\lambda_{1},\lambda_{2},\ldots ,\lambda_{n}$ such that the following condition holds

3.19 $$(E^{n_{1}}-\mu_{1}I)(E^{n_{2}}-\mu_{2}I)\ldots (E^{n_{p}}-\mu_{p}I)=0,$$

where $\mu_{j}$ are polynomials and $n_{1}+n_{2}+\ldots n_{p}=n$.

#### (iii) Moiseev class

Moiseev’s method [121,122] is applied to class of matrices similar to that of Jones type which allow non-algebraic growth at infinity

3.20 $$G(\alpha )=\sum_{m=0}^{n-1}a_{m}(\alpha )E^{m}(\alpha ),$$

where $a_{m}(\alpha )$ are scalar functions and E(α) is a polynomial matrix. In the simplest case of matrix E having distinct eigenvalues almost everywhere, E can be decomposed as

3.21 $$E=T\Lambda T^{-1},$$

where T is the matrix of the eigenvectors and Λ is a diagonal matrix composed of the eigenvalues. Both T and Λ are algebraic matrices (matrices with entries being algebraic functions), and in [121] the Riemann surface R was introduced on which T and Λ are single valued. Further, the matrix factorization problem reduced to a scalar Riemann–Hilbert boundary value problem on R. This problem can be solved in terms of Abelian integrals with the help of Jacobi’s inversion problem as stated by Zverovich in [123]. So, the solution to the problem of factorization of (3.20) is known at least formally, and it possibly can be used for practical purposes. In some special cases, this scheme was realized in a series of articles by Antipov and Silvestrov, and the solution to the factorization problem (as well as to the corresponding problems arising in application) was constructed in an explicit form in terms of special functions (see [124,125] and references therein). The idea of reducing a matrix factorization to a scalar one on a Riemann surface is also developed in [126].

In [127], a simplified method for treating matrices of Moiseev’s type is proposed. An additional restriction is that $a_{m}(\alpha )$ are algebraic functions. The factorization problem is transformed by Hurd’s procedure [49,128,129] into a Riemann–Hilbert boundary value problem on a set of cuts. The method relies on formulation of ordinary differential equation with an unknown coefficient. It is shown that the proposed procedure in the Khrapkov case is equivalent to the standard solution method.

### (f) Factorization of matrices with exponential terms in the entries

#### (i) Integral equation on a finite interval

Consider the system of the convolution equations on a finite interval

3.22 $$\varphi (t)+\int_{0}^{a}k_{0}(t-s)\varphi (s)\text{d}s=f(t),\;0<t<a,$$

note the similarity and differences with the basic integral Wiener–Hopf equation (1.1). The application of the Fourier transform leads to the necessity to factorize the 2n×2n matrix function of type

3.23 $$A(z)=\left(\begin{array}{cc} -{\text{e}}^{-\text{i}az}K(z) & -I+K(z)\\ I+K(z) & -{\text{e}}^{\text{i}az}K(z) \end{array}\right),$$

where the n×n matrix K(z) is the Fourier transform of any extension k onto the whole real line R of the kernel $k_{0}$.

The factorization of matrix (3.23) is studied in [130] under condition that the operator

3.24 $$(B\varphi )(t):=\varphi (t)+\int_{0}^{a}k_{0}(t-s)\varphi (s)\text{d}s,$$

is invertible in $L_{n\times 1}(0,a)$ and it is *a priori* known that partial indices of the matrix A(λ) are equal to zero. In this case, the corresponding factors are found explicitly.

#### (ii) A system of integral equations with an exponential kernel

In [131], the following system of two convolution-type equations on the real semi-axes is considered:

3.25 $$u(x)=\int_{0}^{\infty }k(x-t)u(t)\text{d}t+f(x),\;x\in R_{+},$$

where the kernel k is the following matrix:

3.26 $$k_{b}(x)=\left(\begin{array}{cc} {\text{e}}^{-|x|} & {\text{e}}^{-|x-b|}\\ {\text{e}}^{-|x+b|} & {\text{e}}^{-|x|} \end{array}\right).$$

Fourier transformation of this system leads to a functional equation with matrix kernel

3.27 $$K_{b}(\alpha )=\left(\begin{array}{cc} 2\lambda -1-\alpha^{2} & 2\lambda {\text{e}}^{\text{i}b\alpha }\\ 2\lambda {\text{e}}^{-\text{i}b\alpha } & 2\lambda -1-\alpha^{2} \end{array}\right).$$

Factorization of matrices of such type, as well as some other matrices involving exponential elements, is given in [131].

#### (iii) AKM approach

The name of this approach is simply an abbreviation of the authors’ names in [132] which considers factorization of 2×2 non-rational matrix functions of the form

3.28 $$G(t;k)=\left(\begin{array}{cc} T(t) & -R(t){\text{e}}^{2\text{i}kt}\\ -L(t){\text{e}}^{-2\text{i}kt} & T(t) \end{array}\right),\;t\in R,$$

for an arbitrary real parameter k. Matrices of this type arise as (modified) scattering matrices for the one-dimensional Schrödinger equation and some related Schrödinger-type equations. For the above discussion, the so-called scattering matrix

3.29 $$S(t)=\left(\begin{array}{cc} T(t) & R(t)\\ L(t) & T(t) \end{array}\right),$$

plays an important role. Here T is called the transmission coefficient, and R,L are reflection coefficients from the right and from the left, respectively.

It is supposed that the following conditions are satisfied

(i) T(z)≠0, $z\in \bar{\Pi^{+}}\setminus \{0\}=\{z\in C:\text{Im}\;z\geq 0,z\neq 0\}$, is meromorphic in $\Pi^{+}$ with continuous boundary values on the extended real line, either T(0)≠0 or T(t) is vanishing linearly at t=0, and T(∞)=1.
(ii) R(z) and L(z) are meromorphic on $\Pi^{+}$ with continuous boundary values on the extended real line and vanishes as z→∞ in $\bar{\Pi^{+}}$.
(iii) $G{\left(t;k\right)}^{-1}=QG(-t;k)Q$ for all t∈R, where $Q=\left(\begin{array}{cc} 0 & 1\\ 1 & 0 \end{array}\right)$.
(iv) G(t;k), as the function of t∈R, belongs to a suitable Banach algebra of 2×2 matrix functions within which factorization is possible. This may be the Wiener algebra $W=W^{2\times 2}$ or algebra of functions f(t) such that $f^{*}(\xi )=f(i(\left(1+\xi \right)/\left(1-\xi \right)))\in H_{\alpha }(T),\alpha \in (0,1)$ (which we denote $H_{\alpha }^{2\times 2}$).

First the following statement was proved: *let us consider*

3.30 $$W(t)=\left(\begin{array}{cc} 1 & q(t)\\ -\bar{q(t)} & 1 \end{array}\right),\;t\in R,$$

where q(∞)=0, and either q∈W or $q\in H_{\alpha }(T),\alpha \in (0,1)$. Then W(t) has a unique (right) canonical factorization

$$W(t)=W_{-}(t)W_{+}(t),\;t\in R,$$

where either $W_{\pm }\in W^{2\times 2}$ or $W_{\pm }\in H_{\alpha }^{2\times 2}$, and $W_{\pm }(\infty )=E_{2}$.

It leads to the following result: let matrix (3.28) be a unitary matrix for all t∈R satisfying the following conditions:

(i) T(t)≠0 for all t∈R,
(ii) T(t) can be continued to a meromorphic function on $\Pi^{+}$ with continuous boundary values on $\bar{R}$, and T(∞)=1.
(iii) T(t), R(t) and L(t) belong to either W or to $H_{\alpha },0<\alpha <1$.

Then G(t;k) has a (right) factorization with equal partial indices $\kappa_{1}=\kappa_{2}=\kappa$, where κ is the number of zeros minus the number of poles of T(z) in $\Pi^{+}$.

The factorization has the form

$$G(t;k)=W_{-}(t,k)T_{-}(t)\text{diag}\;\left\{{\left(\frac{t-i}{t+i}\right)}^{\kappa },{\left(\frac{t-i}{t+i}\right)}^{\kappa }\right\}T_{+}(t)W_{+}(t,k),$$

where $W_{-},W_{+}$ are factors of the canonical decomposition of the matrix G(t;k)/T(t) and $T_{-},T_{+}$ are product factors of the scalar function T(t):

$$T(t)=T_{-}(t){\left(\frac{t-i}{t+i}\right)}^{\kappa }T_{+}(t).$$

Some approximate methods for matrices with exponential entries are considered in §4(c).

### (g) Miscellaneous classes

#### (i) EF algorithm

An algorithm based on the successive solution of the scalar boundary value problems was proposed by Feldman *et al.* [23]. They construct the solution of the factorization problem (2.13) when the curve L is either the unit circle T or the real line R.

In the case L=T the 2×2 matrix investigated is a non-singular matrix of the following type

3.31 $$G(t)=\left(\begin{array}{cc} 1 & b(t)\\ c(t) & d(t) \end{array}\right),\;t\in T,$$

satisfying the following conditions:

(i) b,c,d belong to the Wiener algebra W(T) of absolutely converging Fourier series on T;
(ii) b(t)=p(t)/q(t), where $p\in W_{+}(T)$ (i.e. is a Fourier series with vanishing coefficients of negative indices), and q is a polynomial $q(t)=\prod_{j=1}^{k}{\left(t-a_{j}\right)}^{m_{j}}$ with distinct zeros $a_{j}$ lying in the unit disc, $|a_{j}|<1$.

In the case L=R, the Wiener algebra W(R) (and its subalgebras $W_{+}(R)$, $W_{-}(R)$) of the functions ϕ(λ),−∞<λ<+∞, are defined in the standard way

3.32 $$\begin{array}{rl} & W(R)∋\phi (\lambda )=c+\int_{-\infty }^{+\infty }\varphi (\tau ){\text{e}}^{\text{i}\lambda \tau }\text{d}\tau ,\\ & W_{+}(R)∋\phi (\lambda )=c+\int_{0}^{+\infty }\varphi (\tau ){\text{e}}^{\text{i}\lambda \tau }\text{d}\tau \\ \text{and}\; & W_{-}(R)∋\phi (\lambda )=c+\int_{-\infty }^{0}\varphi (\tau ){\text{e}}^{\text{i}\lambda \tau }\text{d}\tau , \end{array}\}$$

where c is an arbitrary complex constant and $\varphi \in L_{1}$ on its domain of integration.

The matrix under consideration is the following:

3.33 $$G(t)=\left(\begin{array}{cc} a(t) & b(t)\\ c(t) & 1 \end{array}\right),\;t\in R,$$

with the corresponding conditions

(i) a,b,c belong to the Wiener algebra W(R);
(ii) b(t)=p(t)/q(t), where $p\in W_{+}(R)$, and q is a rational function $q(t)=\prod_{j=1}^{k}{\left(\left(t-a_{j}\right)/\left(t+\text{i}\gamma_{j}\right)\right)}^{m_{j}}$ with distinct zeros $a_{j}$ laying in the upper half-plane, i.e. $\text{Im}\;a_{j}>0$, and $\gamma_{j}>0$.

In [133], this algorithm is applied to the solution of two problems: (a) the factorization of matrix (3.31) with ratio f(t)=p(t)/q(t) being meromorphically extended either inside the unit disc or outside of it; (b) the factorization of the matrix (3.30) with q∈W(T) and $q^{2}$ being a rational function free of zeros and poles on T.

#### (ii) Linear-fractional problem approach

In [134], a constructive method of factorization was proposed, based on the relation of the homogeneous Riemann–Hilbert boundary value problem (2.15) and the so-called linear-fractional problem.

Consider a homogeneous Riemann–Hilbert boundary value problem

3.34 $$W^{+}(t)=G(t)W^{-}(t),\;t\in L,\;0\in \text{int}\;L,\;\infty \in \text{ext}\;L,$$

with a non-singular Hölder continuous 2×2 matrix coefficient

3.35 $$G(t)=\left(\begin{array}{cc} g_{11}(t) & g_{12}(t)\\ g_{21}(t) & g_{22}(t) \end{array}\right).$$

Denote by $W^{+}(z)={\left(w_{1}^{+}(z),w_{2}^{+}(z)\right)}^{\text{T}}$, $W^{-}(z)={\left(w_{1}^{-}(z),w_{2}^{-}(z)\right)}^{\text{T}}$ the components of the solution to (3.34) and introduce the following functions:

3.36 $$\Phi^{+}(z)=\frac{w_{2}^{+}(z)}{w_{1}^{+}(z)}\;\text{and}\;\Phi^{-}(z)=\frac{w_{2}^{-}(z)}{w_{1}^{-}(z)}.$$

These functions are the components of a piece-wise meromorphic solution to a so-called linear-fractional boundary value problem

3.37 $$g_{11}(t)\Phi^{+}(t)-g_{22}(t)\Phi^{-}(t)+g_{12}(t)\Phi^{+}(t)\Phi^{-}(t)=g_{21}(t).$$

The approach in [134] (see also [135]) is based on different possibilities arising from the study of solvability and representation of the solution to (3.37). These situations are listed in [134,135]. The representation of the solution to the corresponding factorization problem is given for each special case. In [136], this approach is applied to the study of the Riemann–Hilbert boundary value problem with a 3×3 matrix coefficient G(t).

### (h) Rawlins–Williams class of matrix Wiener–Hopf problems

In acoustics and electromagnetism, the matrix kernel A(α) sometimes is a function only of $\gamma (\alpha )={\left(k^{2}-\alpha^{2}\right)}^{1/2}$. This motivates the class considered by Rawlins & Williams in [137]

3.38 $$A(\alpha )=\left(\begin{array}{cc} F(\gamma ) & G(\gamma )F(\gamma )\\ H(\gamma ) & -G(\gamma )H(\gamma ) \end{array}\right),$$

where F,G and H are analytic functions (except possibly when γ=0).

The way that this class of matrices is solved is by first transforming them to a matrix Riemann–Hilbert problem on a half line (along a cut emanating from one of the branch-points of γ(α)). It then transpires that this system can be decoupled into two scalar Riemann–Hilbert problems by Hurd’s method [128], which can be solved explicitly.

Another class of matrices was considered by Jones in [138]:

3.39 $$\text{C}=\left(\begin{array}{cc} f & ge_{1}\\ ge_{2} & f-2ge_{3} \end{array}\right),$$

where f(s), g(s) are analytic in the strip and $e_{1}$, $e_{2}$ and $e_{3}$ are analytic functions in the whole complex plane. A commutative factorization is constructed. There are also extensions to non-commutative factorization by pre-multiplying by analytic matrices. Although it is not obvious, the class of matrices considered by Rawlins and Williams can, in fact, be reduced to Jones’ class [138].

For a review of other classes which originate from applications [49].

## 4. Approximate procedures

Owing to the lack of a general exact constructive factorization procedure, there has been considerable interest in approximate methods for Wiener–Hopf matrix factorization. Numerous approaches have been proposed in the literature [40,68,93,114,127,131,139–148]. We describe some of the more widely applicable classes here. Most approximate constructive methods rely on approximating a Wiener–Hopf problem by a class where exact constructive methods exist. There are also methods which reduce the Wiener–Hopf problem to a different equation such as Fredholm integral equation [94,95,140] but this is outside the scope of this survey.

Suppose there are two functions

4.1 $$K(\alpha )=K_{-}(\alpha )K_{+}(\alpha )\;\text{and}\;\bar{K}(\alpha )={\bar{K}}_{-}(\alpha ){\bar{K}}_{+}(\alpha ).$$

**Question 4.1.** Is it true that if $\bar{K}(\alpha )$ approximates K(α) than ${\bar{K}}_{\pm }(\alpha )$ approximates $K_{\pm }(\alpha )$?

As stated the question is too vague to have a definitive answer, but nevertheless this kind of question motivates the search for approximate solutions. For scalar functions and $L_{p}$ norm if $|K(\alpha )-\bar{K}(\alpha ){|}_{p}\leq \epsilon_{p}$ there are bounds for $|K_{\pm }(\alpha )-{\bar{K}}_{\pm }(\alpha ){|}_{p}$ in terms of the $\epsilon_{p}$ and computable quantities of K(α) [141]. The general answer for a matrix valued function K(α) is negative. This can be the case due to a choice of norm or it can be due to an instability.

The simplest example of instability is obtained by mapping an example [9] from the unit circle to the real line. Consider a diagonal matrix function with partial indices [1, −1].

4.2 $$\left(\begin{array}{cc} \frac{t-i}{t+i} & 0\\ 0 & \frac{t+i}{t-i} \end{array}\right)=\text{I}\left(\begin{array}{cc} \frac{t-i}{t+i} & 0\\ 0 & \frac{t+i}{t-i} \end{array}\right)\text{I}.$$

Perturbing the matrix we have

4.3 $$\left(\begin{array}{cc} \frac{t-i}{t+i} & 0\\ \epsilon & \frac{t+i}{t-i} \end{array}\right)=\left(\begin{array}{cc} 1 & \frac{t-i}{t+i}\\ 0 & \epsilon \end{array}\right)\text{I}\left(\begin{array}{cc} 0 & -1/\epsilon \\ 1 & \frac{t+i}{\epsilon (t-i)} \end{array}\right).$$

This example demonstrates that a small perturbation can change the factors by an arbitrary amount and can also change the partial indices (from {1,−1} to {0,0}). This is significant because the partial indices are uniquely defined. Note that the sum of the partial indices remains the same; this is true in general, by considering the determinant of both sides it is reduced to scalar factorization. For scalar factorization of function f the index is the winding number of the curve (Re f(t),Im f(t)) t∈R hence is stable under perturbations. The partial indices are linked to the growth at infinity of the Wiener–Hopf factorization [149].

The following surprising theorem provides the necessary and sufficient conditions for the partial indexes to remain the same under small enough perturbations.

**Theorem 4.2. Gohberg–Krein [7]**

*The system* $\kappa_{1}\geq \cdots \geq \kappa_{n}$ *of partial indices is stable if and only if*:

4.4 $$\kappa_{1}-\kappa_{n}\leq 1.$$

In fact, this condition is enough to ensure the stability of factors in the Wiener norm.

**Theorem 4.3. Shubin [7]**

*Assume the matrix function* G *to have a Wiener–Hopf factorization and the tuple of its partial indices to be stable. Then, for every* ϵ>0 *there exists a* δ>0 *such that, for* ||F−G||<δ, *the matrix function* F *admits a factorization in which* $||F_{\pm }-G_{\pm }||<\epsilon$.

An obstacle in using this result in applications is that one cannot in general determine the partial indices without constructing the factorization. Thus, in general it is impossible to prove that some approximate factorization of a matrix function is a good approximation. There are some specific results for Khrapkov–Daniele matrix functions [148] and for small perturbations to the identity matrix [145]. This will be discussed in more detail in the sections below. Interestingly, all three methods below reduce the matrix factorization problem to a series of scalar additive Wiener–Hopf splittings. This, in some sense, avoids problems with instabilities of matrix factorization with unstable indices.

### (a) Rational approximation

It is attractive to consider a rational matrix $\bar{K}(\alpha )$ in (4.1) since its factorization can be computed exactly (§3(a)), without the need for a Cauchy-type integral (2.12) representation. Approximate rational solutions were considered early on [150] but they were mainly constructed using *ad hoc* observations ([17], ch. 4.5). In 2000, a systematic way of approximating the Wiener–Hopf equations was developed using a two point Padé approximation (the two points being 0 and ∞ on the real axis) [151]. The power of the method came from the fact that not the whole matrix was approximated but only specific parts and then pole removal method was applied (§3(a)). Since then it proved popular and found applications in different branches of mathematics [93,152,153] including finance [51].

From the last paragraph, it is clear that it is desirable to approximate a matrix function, the whole or various parts, by rational functions $\bar{K}(\alpha )$. A natural question to ask: which K(α) can be approximated by rational matrix functions $\bar{K}(\alpha )$? For this question, we have to specify in what sense are K(α) and $\bar{K}(\alpha )$ close. It is not an easy task to decide what the correct norm is to consider, but good candidates are $L_{p}$ norms or Sobolev spaces [154]. We will review some known facts about approximation by rational functions, mostly by Padé approximants. Functions with branch cuts (multi-valued) cannot be ‘fully’ approximated by rational functions which are single-valued. But for a function analytic at infinity the next best result is true, the maximal domain of K(α) where the function has a single-valued branch is the domain of convergence of the diagonal Padé approximants for K(α). In fact, most of the poles tend to the boundary of the domain of convergence and lie on the system of cuts that makes the function single-valued [155,156]. Numerical experimentation has confirmed this holds true for even small degree of rational approximation and, what is more, often the interlacing of poles and zeros occurs on the branch cut [141,157]. This simulates the behaviour of the branch cut; the values on either side of the cut made by poles and zeros has a jump that tends to the exact value as the rational approximation degree is increased [158].

The practical implementation of the rational approximations has now been well developed. In particular there are numerous algorithms on *Chebfun* (Matlab). One of the latest additions in *Chebfun* is the very fast AAA rational approximation [159]. It is also useful to construct a mapping of the real line to the unit interval since most existing algorithms are for bounded intervals [141]. The main difficulty with rational approximations arise when a branch cut goes though infinity as in $\gamma (\alpha )=\sqrt{\alpha^{2}-k^{2}}$ (which is frequently encountered in diffraction problems). The problem arises because the real line crosses the branch cut at infinity. Hence rational approximation can no longer be accurate on the whole contour (the real line). It can still be very accurate on a bounded domain and hence rational approximations of γ(α) are used in acoustics far-fieldcalculations [144].

### (b) Asymptotic Wiener–Hopf factorization

Let M be a matrix function analytic around the real axis R. Let I be the identity matrix and ε∈[0,1]. We can write

4.5 M(x)=I+εG(x),

and then the asymptotic algorithm produces a good approximate Wiener–Hopf factorization if εG(x) is small compared to I [145,147].

Asymptotic Wiener–Hopf factorization looks for an approximate factorization of the form

4.6 $$I+\varepsilon G\approx (I+\sum_{i\in N^{*}}\varepsilon^{i}G_{i-1}^{-})(I+\sum_{i\in N^{*}}\varepsilon^{i}G_{i-1}^{+}).$$

We define the error $\Delta_{j}$ at a step j as the term neglected in the approximation:

4.7 $$\Delta_{j}=I+\varepsilon G-S_{j}^{-}S_{j}^{+},\;\text{where}\;S_{j}^{-}=I+\sum_{i=1}^{j}\varepsilon^{i}\;G_{i-1}^{-},\;\text{and}\;S_{j}^{+}=I+\sum_{i=1}^{j}\varepsilon^{i}\;G_{i-1}^{+}.$$

For the first step, we are looking for $G_{0}^{-}$ and $G_{0}^{+}$, respectively, analytic on the lower half-plane and the upper half-plane, so that

$$\begin{array}{rl} I+\varepsilon G & \approx (I+\varepsilon G_{0}^{-})(I+\varepsilon G_{0}^{+}),\\ & =I+\varepsilon (G_{0}^{-}+G_{0}^{+})+\varepsilon^{2}(G_{0}^{-}G_{0}^{+}). \end{array}$$

Hence, at the first order of ε, we have the Wiener–Hopf additive splitting:

4.8 $$G=G_{0}^{-}+G_{0}^{+}.$$

The error is

4.9 $$\Delta_{1}=(I+\varepsilon G)-(I+\varepsilon G_{0}^{-})(I+\varepsilon G_{0}^{+})=\varepsilon^{2}G_{0}^{-}G_{0}^{+}.$$

For the second order of ε:

$$\begin{array}{rl} I+\varepsilon G & \approx (I+\varepsilon G_{0}^{-}+\varepsilon^{2}G_{1}^{-})(I+\varepsilon G_{0}^{+}+\varepsilon^{2}G_{1}^{+}),\\ & =I+\varepsilon (G_{0}^{-}+G_{0}^{+})+\varepsilon^{2}(G_{1}^{-}+G_{1}^{+}+G_{0}^{-}G_{0}^{+})+\varepsilon^{3}(G_{1}^{-}G_{0}^{+}+G_{0}^{-}G_{1}^{+})+\varepsilon^{4}G_{1}^{-}G_{1}^{+}, \end{array}$$

where the term in $\varepsilon^{2}$ should be equal to zero. Therefore, we can deduce $G_{1}^{-}$, $G_{1}^{+}$ and thus $\Delta_{2}$:

4.10 $$G_{1}=G_{1}^{-}+G_{1}^{+}=-G_{0}^{-}G_{0}^{+},\;\Delta_{2}=\varepsilon^{3}(G_{1}^{-}G_{0}^{+}+G_{0}^{-}G_{1}^{+})+\varepsilon^{4}\;G_{1}^{-}G_{1}^{+}.$$

In the same manner, arbitrary order approximations are constructed and, under some conditions, its convergence can be established [145] and used in [62] for the factorization on the unit circle. Furthermore, this technique can be extended for the case of a set of stable partial indices [40] or, in some cases, even for unstable sets [160].

### (c) Neglecting coupling and iteration

The main difficulty in solving matrix Wiener–Hopf equation arises from the fact that there are *coupled* scalar equations. In some situations the coupling is small and a reasonable approximation can be obtained by neglecting it. In other words the original matrix is close to being diagonal or triangular. More often the coupling is too strong to give a desired approximation see Section 4.4 in [17] or [161], nevertheless the same ideas can still be used. One way is through the use of iterative methods, which in the context of Wiener–Hopf equations were proposed early on [162,163]. One of the reasons that they did not gain popularity in the past is that they are computationally intensive. But recent advances in computing Cauchy-type transforms, and significant computational resource, have now made it a practical approach [96,164,165]. In particular, it has been applied to a class of triangular matrix functions containing exponential factors [166]. The aim is to find functions $\Phi_{-}^{\left(0\right)}(\alpha )$, $\Phi_{-}^{\left(L\right)}(\alpha )$, $\Psi_{+}^{\left(0\right)}(\alpha )$ and $\Psi_{+}^{\left(L\right)}(\alpha )$ analytic in respective half-planes, satisfying the following relationship:

4.11 $$\left(\begin{array}{c} \Phi_{-}^{\left(0\right)}(\alpha )\\ \Phi_{-}^{\left(L\right)}(\alpha ) \end{array}\right)=\left(\begin{array}{cc} A(\alpha ) & B(\alpha ){\text{e}}^{\text{i}\alpha L}\\ C(\alpha ){\text{e}}^{-\text{i}\alpha L} & 0 \end{array}\right)\left(\begin{array}{c} \Psi_{+}^{\left(0\right)}(\alpha )\\ \Psi_{+}^{\left(L\right)}(\alpha ) \end{array}\right)+\left(\begin{array}{c} f_{1}(\alpha )\\ f_{2}(\alpha ) \end{array}\right),$$

on the strip H. The functions A(α), B(α) and C(α) are known and L is a positive constant. The method has found applications in aeroacoustics [167], crack propagation problems [168]. It has been extended to n-dimensional matrix problems and applied to scattering from multiple co-linear plates [169]. We would also like to note that there is a similarity of the above iteration methods with a concept in diffraction called the Schwarzschild series [170–173]. This illustrates a useful viewpoint: many problems which give rise to a matrix Wiener–Hopf equation can be thought of as an interaction of a number of distinct problems, each of which would lead to a scalar Wiener–Hopf equation [174].

## 5. Related methods and open problems

To solve Wiener–Hopf-type problems which arise in applications, the following are desirable

(i) A systematic way of determining if the posed problem is of Wiener–Hopf-type and has a unique solution.
(ii) A justified and executable algorithm capable of verifying whether a given matrix possesses a canonical factorization (all partial indices are equal to zero) or not.
(iii) A routine method of deriving and transforming the original problem into a Wiener–Hopf equation with regularized matrix kernel (allowing canonical factorization).
(iv) Criteria for determining which class of matrix Wiener–Hopf equation the given equation belongs to.
(v) A unified constructive method for an exact or an approximate factorization.

For problems of scalar Wiener–Hopf-type, the above points can be considered relatively complete. In the case of a classical matrix Wiener–Hopf equations the first one is straightforward while the remaining points continue to offer future avenues of research. It is possible to develop alternative methods for solving equations of Wiener–Hopf-type, for example based on Fredholm integral equation theory [140,175,176], and also more theoretical operator based approaches to convolution integral equations [154,177].

There are equations of Wiener–Hopf type which have not been reviewed here. For example, PDEs on wedge domains are not immediately in a standard class, but can be stated as generalized Wiener–Hopf equations if appropriate transformations/mapping are considered [178,179]. The difficulty with wedge domains is that the Fourier transform is not the natural operator to use, hence a mapping is required. Alternatively, the Mellin transform [180] could be employed if the respective differential operators are of the same homogeneity, and a combination of the Mellin and Fourier transform could be used for multilayered-multiwedged domains [27,181,182]. There is also a link to the unified transform method which provides a systematic way of deriving a natural transform for some polygonal domains [71]. A natural but difficult generalization is to consider (1.1) in more variables (a double integral) [183,184] leading to interesting Wiener–Hopf related methods in multi-variate complex analysis [185,186]. A different direction is when the kernel (1.1) has support on the half-line but is not of convolution type (the equation is referred to as being of Hammerstein type) [187]. There is also a wealth of literature on spectral properties of Toeplitz matrices/operators [188–191] which is a related topic but outside the scope of this review.

Interest in Wiener–Hopf techniques has been steady ever since the 1970s, judging by the number of papers containing ‘Wiener–Hopf’ on *mathscinet*, with a total around 3000 to date (it is likely to be a significant underestimate since many of the references for this review either do not contain ‘Wiener–Hopf’ in the title, or are published in engineering and physics journals). The related Riemann–Hilbert boundary value method has also been popular with around 4,600 publications; with a small but steady increase in number of publication each year overall. The Wiener–Hopf method could be viewed through many lenses, has many related problems, arises in numerous applications and still has many interesting open problems. It can be expected that further work in coming years will resolve some of the long-standing questions related to, and limitations of, the Wiener–Hopf method, and hence extend its relevance and applicability still further.

## Footnotes

1 In fact, in the original paper [1] the Laplace transform was applied and the corresponding functional equation is valid in a vertical strip. Interestingly the original paper contained a mistake; it claimed that $\gamma^{n}(z)={\left(z^{2}-1\right)}^{n/2}\to z^{n}$ as z→±∞ where n was the number of zeros of the kernel in the strip of analyticity (this is only true for n even). This was pointed out in ([3], ch. 16), and resulted from an incorrect choice of branch cut. However, it did not influence the actual results they obtained in the second part of the paper, since they assumed that the kernel is even and hence n is even.

2 For a long time, it was supposed that Plemelj’s work gave a complete answer on the Hilbert question. But in the late 1980s Bolibrukh [4] showed that the proof of Plemelj is incomplete and that the negative answer is also possible. In fact, it was shown that Plemelj’s positive answer for the question is valid for the so-called ‘regular’ [4, p. 7] variant of the Riemann–Hilbert boundary value problem.

3 It would be more historically accurate to call it the Riemann boundary value problem, as still used by Gakhov’s school; however, we will use the title Riemann–Hilbert boundary value problem, introduced by Muskhelishvili, as it is more common nowadays.

4 These formulae are valid for L=R and under weaker assumptions on G,g [18].

5 This is a crucial problem, highly dependent on the properties of the matrix coefficient G, and NOT always possible to be performed in a constructive way.

## Data accessibility

This article has no additional data.
